# Transcriptome Analysis of *Plectropomus leopardus* Erythrocytes Reveals Transcriptional Immune Suppression Induced by *Vibrio harveyi* Infection

**DOI:** 10.3390/ani16182831

**Published:** 2026-09-09

**Authors:** Jingmiao Yang, An Li, Zhenyi An, Xiaowei Zhou, Qiang Chen, Lijuan Zhang, Kai Feng, Fen Zeng, Xiaojuan Chen, Bin Sun

**Affiliations:** 1 Fujian Provincial Key Lab of Coastal Basin Environment, Fujian Polytechnic Normal University, Fuqing 350300, China; yangjm@fpnu.edu.cn; 2 Institute of Biotechnology, Fujian Academy of Agricultural Sciences, Fuzhou 350003, China; 13705945051@139.com (Q.C.); zhanglijuan@faas.cn (L.Z.); 3 Zhejiang Key Laboratory of Coastal Biological Germplasm Resources Conservation and Utilization, Zhejiang Mariculture Research Institute, Wenzhou 325000, China; anliwenzhou@163.com; 4 State Key Laboratory of Mariculture Breeding, Key Laboratory of Marine Biotechnology of Fujian Province, College of Marine Sciences, Fujian Agriculture and Forestry University, Fuzhou 350002, China; q468448923@126.com (Z.A.); zxwdyx1206@163.com (X.Z.); yapilf@outlook.com (K.F.); zf11080321@163.com (F.Z.)

**Keywords:** fish, innate immunity, nucleated erythrocytes, bacterial endocytosis, host–pathogen interaction

## Abstract

Unlike mammalian erythrocytes, teleost erythrocytes are nucleated and transcriptionally active, yet their immune functions remain not fully understood. The leopard coral grouper (*Plectropomus leopardus*) is an economically important marine fish and is highly susceptible to *Vibrio harveyi* infection. In this study, we aim to investigate the immune role of erythrocytes during *V. harveyi* infection. Flow cytometry analysis showed that erythrocytes actively internalize *V. harveyi* in a dose-dependent manner. Transcriptome profiling of erythrocytes from control and *V. harveyi*-infected groups was performed to reveal the host immune responses after bacterial internalization. A total of 5995 differentially expressed genes (DEGs) were identified and significantly enriched in immune-related pathways, with the MAPK signaling pathway, apoptosis, and NF-κB signaling pathway as the three core pathways. Heatmap analysis further revealed transcriptional downregulation of MAPK kinases, apoptotic components, and cytokines (interleukins, chemokines, TNFs, and interferons). These findings provide transcriptional evidence for the immune involvement of teleost erythrocytes and offer insights into host–pathogen interactions during *V. harveyi* infection.

## Simple Summary

Unlike human red blood cells, fish red blood cells have a nucleus and can fight pathogens. The leopard coral grouper is a valuable farmed fish, but it often dies from infections caused by a bacterium called *Vibrio harveyi*, which hurts the fish farming industry. In this study, we found that leopard coral grouper red blood cells can swallow *Vibrio harveyi*. However, *Vibrio harveyi* also suppress the immune defenses of the fish at the transcriptional level by turning down important immune genes. These findings help explain why this fish gets sick so easily and may lead to better ways to protect farmed fish from the bacterial infection.

## 1. Introduction

Erythrocytes constitute the predominant cellular component of blood [1,2,3]. Unlike their mammalian counterparts, which lose their nuclei during maturation, erythrocytes in non-mammalian vertebrates retain both nuclei and organelles, enabling protein synthesis upon stimulation [1]. These nucleated erythrocytes participate actively in host defense, mounting immune responses against a broad spectrum of pathogens including bacteria (e.g., *Escherichia coli* [2], *Candida albicans* [3]) and viruses (e.g., infectious salmon anemia virus [4], piscine orthoreovirus [5], viral hemorrhagic septicemia virus [6], and infectious pancreatic necrosis virus [7]). At the molecular level, nucleated erythrocytes express pattern recognition receptors (PRRs) to detect pathogen-associated molecular patterns (PAMPs) on invading microorganisms [8,9,10]. For example, rainbow trout erythrocytes express toll-like receptor 3 (*TLR3*) and *TLR9*, with *TLR9* expression enhanced by LPS stimulation [8], while Atlantic salmon erythrocytes express *RIG-I* for sensing intracellular viral double-stranded RNA [9]. Following viral hemorrhagic septicemia virus stimulation, rainbow trout erythrocytes also express multiple NOD-like receptor family members including *NOD2*, *NLRX1*, *NLRC3*, and *NLRC5* [10]. PRRs activation of nucleated erythrocytes initiates downstream signaling cascades that drive transcription of innate immune effectors, including interferons, interleukins, chemokines, and antimicrobial peptides [11]. Beyond innate immunity, nucleated erythrocytes exhibit antigen-presenting cell-like properties [12]. For example, teleost erythrocytes can express both major histocompatibility complex class I (*MHC-I*) and class II (*MHC-II*) molecules, which can be recognized by T cells and bridge into adaptive immune responses [12].

It is well known that B cells, macrophages, and neutrophils are professional phagocytes, whereas erythrocytes are not [13]. Nonetheless, non-professional phagocytes, including erythrocytes, retain the capacity to internalize extracellular components [2,3]. Endocytosis is a process that brings extracellular particles into the cell interior, encompassing phagocytosis, clathrin-mediated endocytosis, caveolae-mediated endocytosis, macropinocytosis, pinocytosis, and others [14]. A previous study in flounder (*Paralichthys olivaceus*) demonstrated that erythrocytes exhibited a marked capacity to engulf both live and inactivated *Edwardsiella tarda*, with the uptake of live bacteria occurring via clathrin-mediated endocytosis [15]. Additionally, rainbow trout erythrocytes have been reported to engulf the fungal pathogen *Candida albicans* [3]. However, whether erythrocytes of the leopard coral grouper possess similar endocytic capacity remains unclear and is investigated in this study.

Leopard coral grouper (*Plectropomus leopardus*, *P. leopardus*), a commercially important aquaculture species in China, is highly susceptible to *Vibrio harveyi*, a major bacterial pathogen in intensive farming [16,17,18]. In natural aquaculture environments, *V. harveyi* often co-occurs with other Vibrio species in *P. leopardus*, including *V. owensii* and *V. rotiferianus*, which collectively contribute to polymicrobial infections and disease complexity [16]. Understanding the immune response of erythrocytes to *V. harveyi* thus provides a foundation for addressing multi-pathogen challenges in intensive farming systems. Recent studies have mainly focused on elucidating the interactions between *V. harveyi* and fish tissues to investigate host immune defense [17,18]. For example, a transcriptome analysis of *P. leopardus* revealed *V. harveyi*-induced gene expression profiles in spleen and liver tissues and identified key immune pathways and genes closely linked to *V. harveyi* infection [17]. However, the transcriptome-level changes in blood, particularly in erythrocytes, as the most abundant cellular component, have not been investigated.

In this study, we examined the capacity of *P. leopardus* erythrocytes to internalize *V. harveyi*, and analyzed the transcriptome of erythrocytes induced by *V. harveyi* challenge. We identified a large amount of differentially expressed genes (DEGs) and tested enrichment of Gene Ontology terms (GOs) and Kyoto Encyclopedia of Genes and Genomes (KEGG) pathways of the DEGs. Furthermore, we identified core immune pathways and characterized the gene expression patterns involved in response to *V. harveyi* infection. Our results provided a valuable molecular basis for further investigation of the mechanism of *P. leopardus* erythrocytes against bacterial infection.

## 2. Materials and Methods

### 2.1. Isolating Erythrocytes from the Leopard Coral Grouper

Leopard coral grouper erythrocytes were obtained as described previously [15,19]. In brief, fish were anesthetized with tricaine methane sulfonate (Sigma, St. Louis, MO, USA), and caudal vein blood was collected and diluted with PBS (Solarbio, Beijing, China) containing 10 U/mL heparin (Solarbio, Beijing, China). Erythrocytes were isolated by placing the diluted blood on the top of 1.070 g/mL Percoll (GE Healthcare, Uppsala, Sweden) at 400× *g* for 10 min. To obtain highly purified erythrocytes, the cell suspension was subjected to 2–3 times additional rounds of purification on Percoll gradients, and the purity was examined to be 100% by confocal microscopy (Figure S1). The resulting purified erythrocytes were finally resuspended in RPMI-1640 medium supplemented with 10% calf serum (Gibco, Carlsbad, CA, USA), 100 U/mL penicillin, 100 µg/mL streptomycin, and 50 µg/mL gentamicin (all from Solarbio, Beijing, China).

### 2.2. Bacteria Culture and Preparation

*V. harveyi* used in this study was isolated from diseased fish obtained from Fujian province of China and stored in our laboratory. Bacteria were cultured in Luria–Bertani (LB) medium at 28 °C to an OD_600_ of 0.7. The bacteria were collected by centrifugation at 2000× *g* for 5 min. The bacterial pellet was washed and suspended in PBS. After that, the bacteria were labeled with fluorescein isothiocyanate (FITC) (Tiangen, Beijing, China) as reported previously [15]. The bacterial cells were washed three times with PBS to remove unbound FITC and then adjusted to suitable concentrations. To prepare inactivated bacteria, *V. harveyi* was cultured as above and treated with 0.4% formaldehyde, followed by labeling with FITC as described above.

### 2.3. Flow Cytometry Analysis

To examine the capacity of Leopard coral grouper erythrocytes to uptake *V. harveyi*, the erythrocytes were incubated with FITC-labeled live *V. harveyi* at various multiplicities of infection (MOI = 1:1, 5:1, 10:1, 20:1) for 2 and 4 h. After incubation, the cells were washed three times with PBS to remove unbound bacteria, and the fluorescence of extracellular and surface-attached bacteria was quenched with 0.125% trypan blue prior to flow cytometric analysis, ensuring that the detected signal represented only intracellular bacteria. To compare the uptake of live and inactivated *V. harveyi*, erythrocytes were incubated with FITC-labeled live or inactivated *V. harveyi* at MOI = 20:1 for 4 h. Bacterial uptake was then determined by flow cytometry as described previously [20].

### 2.4. Transcriptome Sequencing

For RNA sequencing library construction and sequencing, erythrocytes from eighteen fish were obtained as described above and divided into two groups. For the infected group, erythrocytes were incubated with *V. harveyi* at MOI = 20:1 for 4 h, and the control group without bacterial treatment. To prepare biological replicates, erythrocytes from every three fish were pooled into a single sample within each group, resulting in six pooled samples in total (three replicates per group). This pooled sample strategy is adopted to characterize the group-level transcriptional response of erythrocytes against *V. harveyi* infection and to obtain sufficient RNA for sequencing, which, to some extent, limits the assessment of individual variation. For transcriptome analysis, six RNA seq libraries were constructed using the pooled samples. Total RNA was extracted from each pooled sample using TRIzol reagent (Invitrogen, Carlsbad, CA, USA) according to the manufacturer’s protocol. RNA quality and integrity were evaluated using an Agilent 2100 Bioanalyzer and agarose gel electrophoresis. Sequencing libraries were constructed following the standard Illumina protocol as previously described [19]. Briefly, mRNA was enriched from total RNA using Oligo(dT) beads (Qiagen, Hilden, Germany), fragmented, and reverse-transcribed into cDNA. The cDNA was then purified, end-repaired, poly(A)-tailed, and ligated to Illumina adapters. Sequencing was performed on an Illumina Novaseq X Plus platform by OmicsMaster Biotechnology Co., Ltd. (Guangzhou, China).

RNA-seq data processing: Raw reads from each library were processed using FASTQ (version 0.20.1) by removing low-quality reads, including those with quality score (Q) ≤ 20, containing adapter sequences, and undetermined nucleotides > 10%. The remaining reads were then aligned to the ribosomal RNA (rRNA) database using Bowtie 2, and the rRNA-mapped reads were discarded. The rRNA-depleted reads were subsequently mapped to the leopard coral grouper reference genome (GenBank accession: YSFRI_Pleo_2.0) using HISAT2. Transcripts were reconstructed with Stringtie (version 2.2.1), and transcript abundance was quantified using RSEM (version 1.3.3). Gene expression levels were normalized using FPKM method as preciously reported [21].

### 2.5. Differentially Expressed Genes (DEGs) Identification, Validation, and Enrichment Analysis

Differential expression analysis was performed using the R package edgeR (version 3.40.2). Pairwise comparisons between the infected and control groups were conducted using the exact negative binomial test. Transcripts with a false discovery rate (FDR) < 0.05 and log2|FC| > 1 were defined as DEGs. To validate the RNA-seq results, the expression of ten DEGs was assessed by quantitative real-time reverse transcription PCR (qRT-PCR) as previously described [22]. Gene primers were designed using the NCBI primer design tool (https://www.ncbi.nlm.nih.gov) and are listed in Table S1. The gene expression levels were calculated using comparative threshold cycle method (2^−ΔΔCT^) with *β-actin* as an internal reference [18]. Functional enrichment of the DEGs was performed using the GO (http://geneontology.org) and KEGG (http://www.genome.jp/kegg/ accessed on 12 February 2026) databases. Significantly enriched GO terms and KEGG pathways were determined by a hypergeometric test with a threshold of *p*-value < 0.05.

### 2.6. Construction of Interaction Network

Immune pathway interaction network was constructed based on the hierarchical relationships in the KEGG database (http://www.genome.jp/kegg/). The interaction degree of each pathway was calculated to evaluate its centrality within the network. DEGs in each core immune pathway were used to construct protein–protein interaction (PPI) networks using the String software (version 12.0) (http://string-db.org/) as reported previously [23].

### 2.7. Statistical Analysis

All experiments were performed three times or in triplicate. Graphical representation and statistical analyses were conducted using GraphPad Prism 6 (https://www.graphpad.com). Comparisons between groups were analyzed by Student’s *t*-test, and statistical significance was defined as *p*-value < 0.05.

## 3. Results

### 3.1. Uptake of V. harveyi by Leopard Coral Grouper Erythrocytes

**Figure 1:**
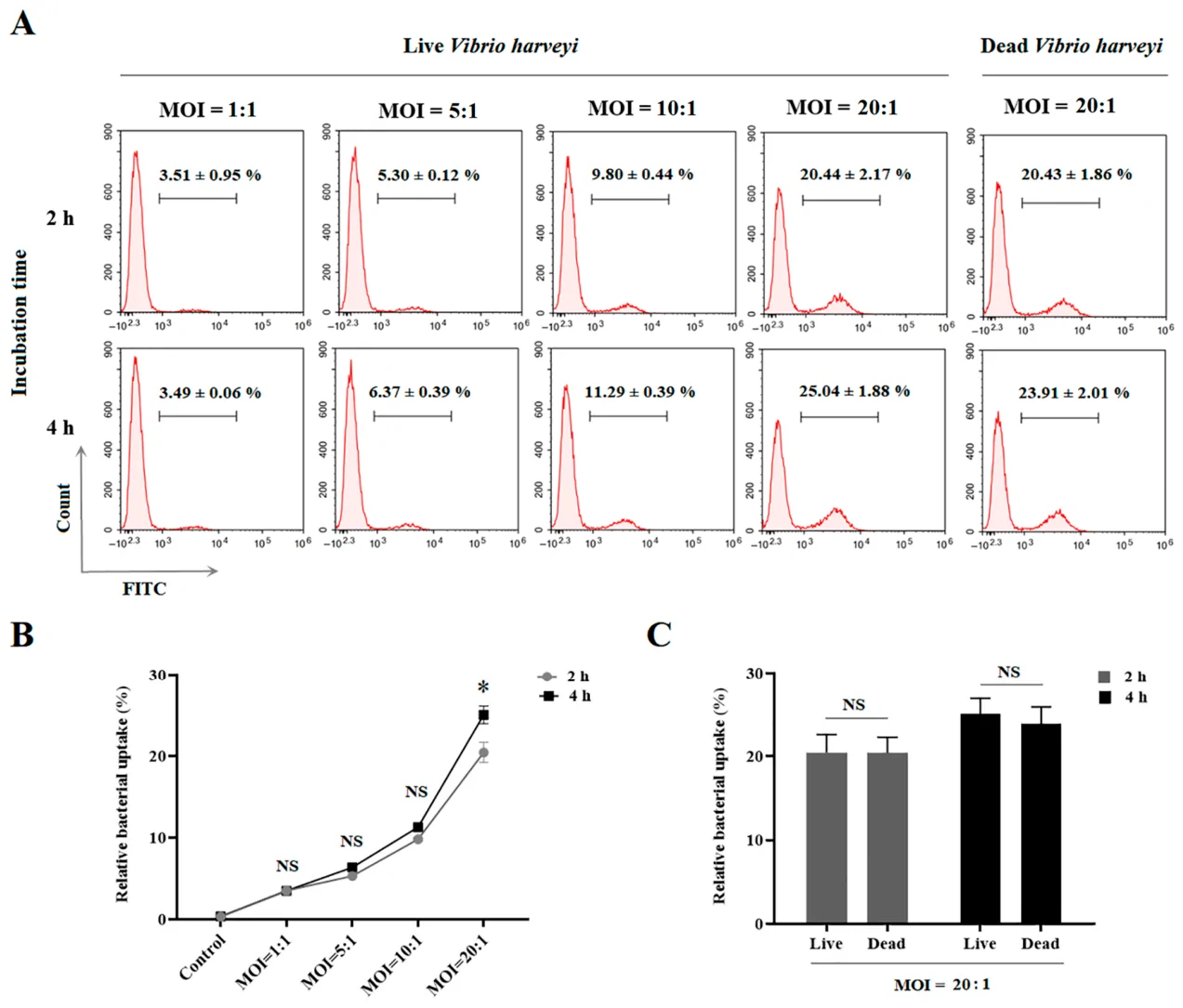
Active endocytosis of *Vibrio harveyi* by erythrocytes of leopard coral grouper (*Plectropomus leopardus*). (**A**) Erythrocytes were incubated with FITC-labeled *V. harveyi* at various multiplicities of infection (MOI) for 2 or 4 h. After quenching of extracellular fluorescence, bacterial uptake was quantified by flow cytometry. (**B**) Rates of live *V. harveyi* uptake by erythrocytes in (**A**). Data are the means of three independent experiments and shown as means ± SEM. Data were analyzed with Student’s *t*-test, and statistical significance was defined as * *p* < 0.05. (**C**) Comparison of erythrocytes uptake between live and dead *V. harveyi* in (**A**). “NS” means no significance.

To examine the capacity of Leopard coral grouper erythrocytes to uptake *V. harveyi*, the cells were incubated with FITC-labeled live *V. harveyi* at various MOI (1:1, 5:1, 10:1, and 20:1) for 2 and 4 h. After removing free unattached bactericides, the fluorescence of extracellular and surface-attached *V. harveyi* was quenched before flow cytometry analysis, ensuring that the detected fluorescence signal reflected only intracellular bacteria ingested by erythrocytes. The results demonstrated a MOI-dependent increase in bacterial uptake at both time points (Figure 1A), with detailed uptake rates summarized in Figure 1B. Notably, when the MOI reached 20, the uptake rate at 4 h was significantly higher than that at 2 h (Figure 1B). To determine whether active ingestion was involved, erythrocytes were incubated with live or dead bacteria at an MOI of 20 for 2 and 4 h. Flow cytometry analysis revealed comparable uptake efficiencies of erythrocytes for both live and dead bacteria (Figure 1C). Collectively, these findings indicated that leopard coral grouper erythrocytes are capable of actively internalizing *V. harveyi* in a dose-responsive manner, and this process does not discriminate between live and dead bacterial cells.

### 3.2. Transcriptome Sequencing and DEGs Identification

**Figure 2:**
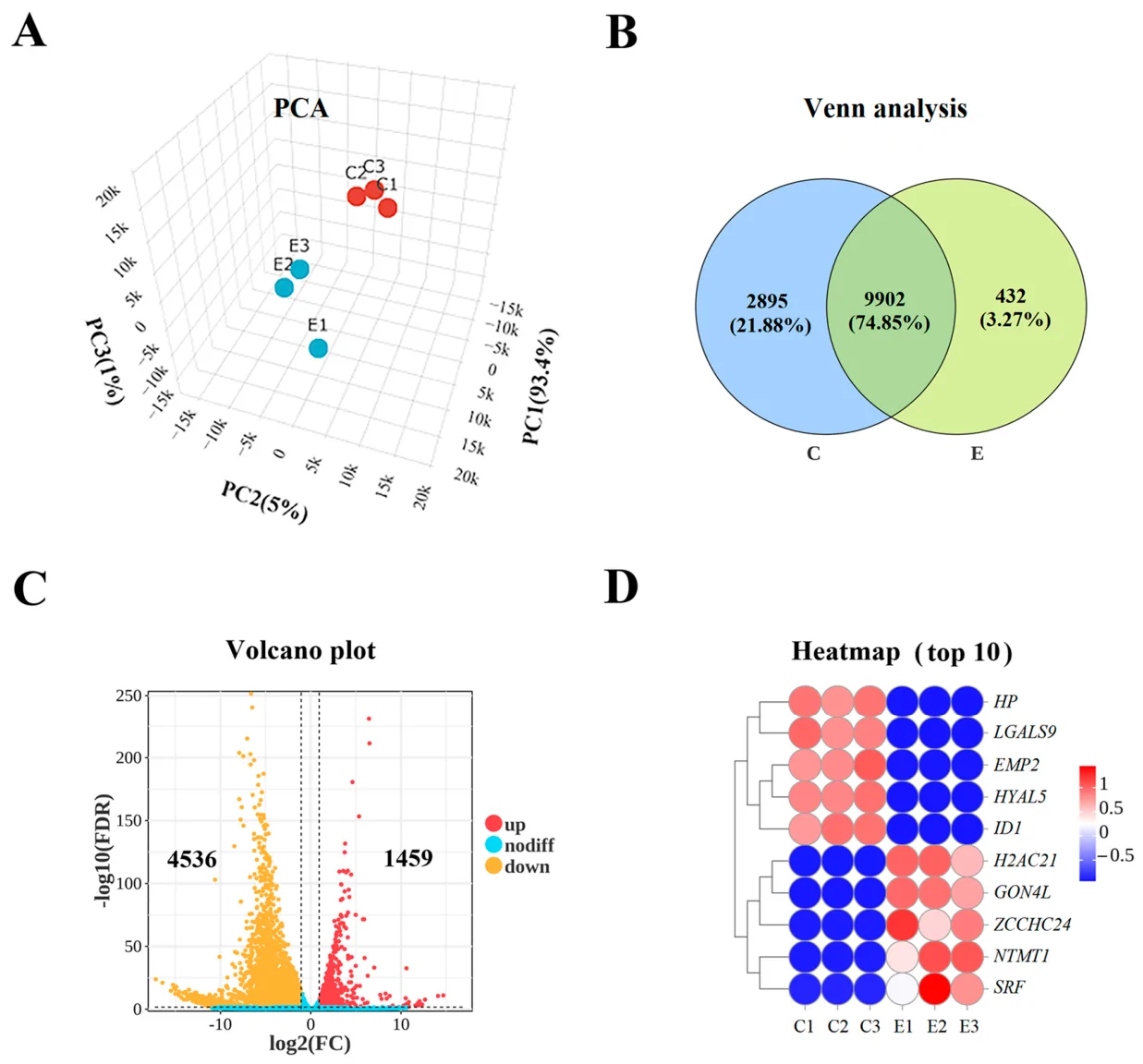
Transcriptome sequencing and differentially expressed genes (DEGs) identification. (**A**) Three-dimensional principal component analysis (PCA) plot showing sample distribution; (**B**) Venn diagram analysis of the identified genes in control and experimental groups; (**C**) volcano plot analysis of DEGs, and the vertical dashed lines indicate the fold-change threshold (log2|FC| = 1); (**D**) the top 10 DEGs were ranked by the absolute value of log_2_ fold change (log_2_|FC|) and displayed via heatmap.

To reveal the transcription profiles of erythrocytes against *V. harveyi* infection, three libraries were constructed using RNA from erythrocytes infected with *V. harveyi* at an MOI of 20 for 4 h. Similarly, three libraries were constructed using RNA from uninfected control erythrocytes. A total of six libraries were sequenced, and the data were summarized in Table 1. An average 47,946,350 raw reads were obtained, 99.43% of which passed the quality filtering process. After removing ribosomal RNAs, a mean number of 47,634,488 filtered clean reads was obtained from each library, and 82.59–87.69% of the clean reads were mapped to the leopard coral grouper genome. The three-dimensional principal component analysis (PCA) plot revealed a clear separation between the control (C) and experimental groups (E) into two distinct clusters, with high reproducibility observed among samples within each group (Figure 2A). A total of 13,229 genes were identified, with 9,902 genes common to both groups, 2,895 unique to the control group, and 432 unique to the experimental group (Figure 2B).

DEGs were identified based on the criteria of gene expression level (log2|FC| > 1) and a false discovery rate (FDR) (FDR < 0.05). Compared to the control group, the *V. harveyi*-infected group exhibited 5995 DEGs, 1459 and 4536 of which were up- and downregulated, respectively. The distributions of the DEGs are shown in Figure 2C, and the expression levels of the top 10 DEGs are displayed in the heatmap (Figure 2D). To validate the DEGs identified by RNA-seq, ten DEGs were randomly selected for qRT-PCR analysis. The results showed that the expression trends of these DEGs were in good agreement with that of RNA-Seq, confirming the reliability of the transcriptomic analysis (Figure S2).

### 3.3. GO and KEGG Enrichment Analysis of the DEGs

**Figure 3:**
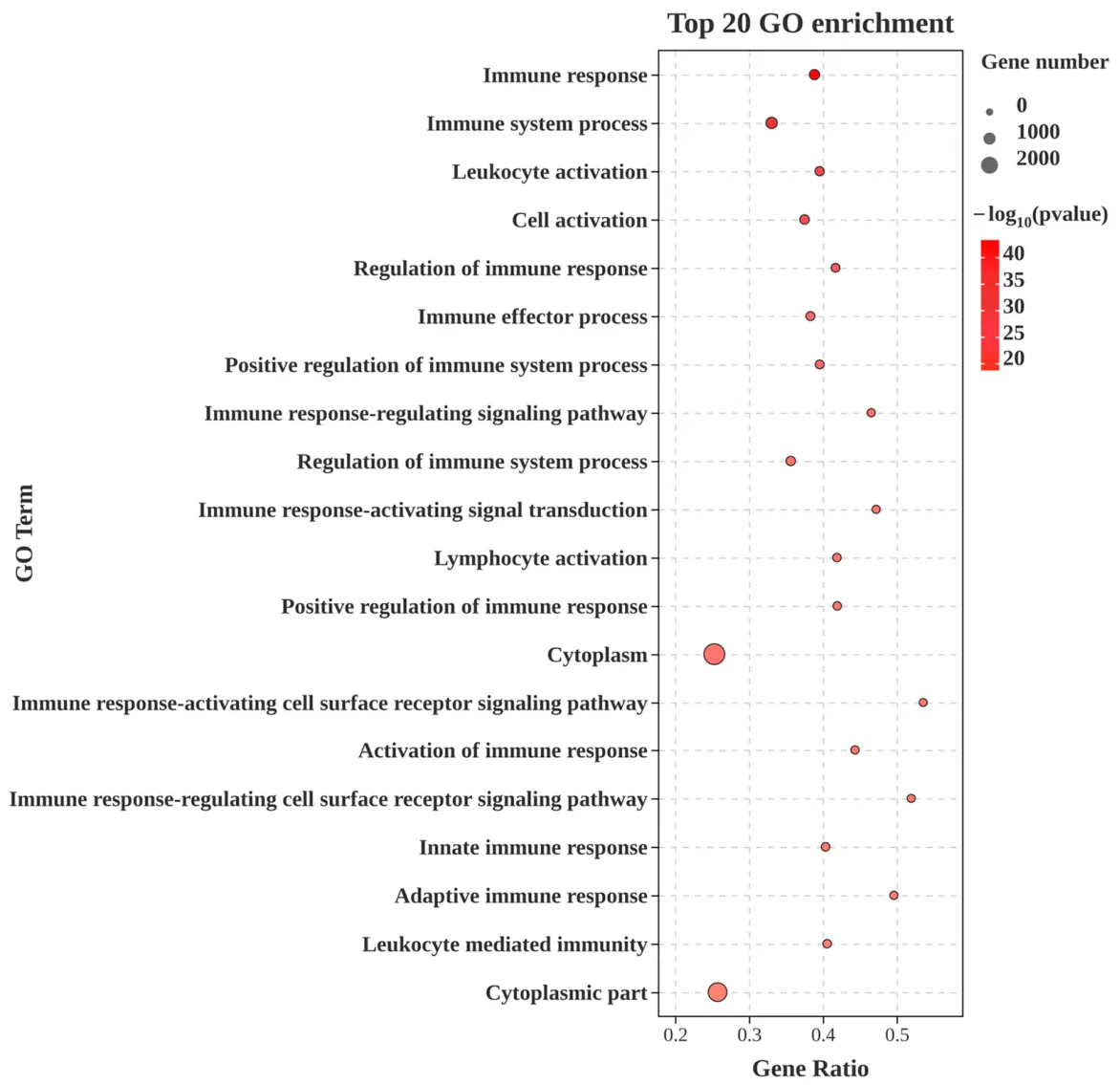
Gene Ontology (GO) enrichment analysis of differentially expressed genes (DEGs). Distribution of the top 20 significantly enriched GO terms (*p*-value < 0.05). Circle size represents the number of enriched DEGs. Color intensity indicates the level of significance.

Based on GO annotation, the 5995 DEGs were categorized into three main categories: biological process (BP), cellular component (CC), and molecular function (MF). Enrichment analysis was then performed to identify significantly over-represented GO terms within each category. The top 20 significantly enriched GO terms are shown in Figure 3. Notably, 90% of these terms were associated with immune functions, encompassing the immune response, immune system process, leukocyte activation, cell activation, regulation of immune response, and immune effector process, among others. The top three GO terms ranked by gene ratio were the immune response-activating cell surface receptor signaling pathway, adaptive immune response, and immune response, respectively.

**Figure 4:**
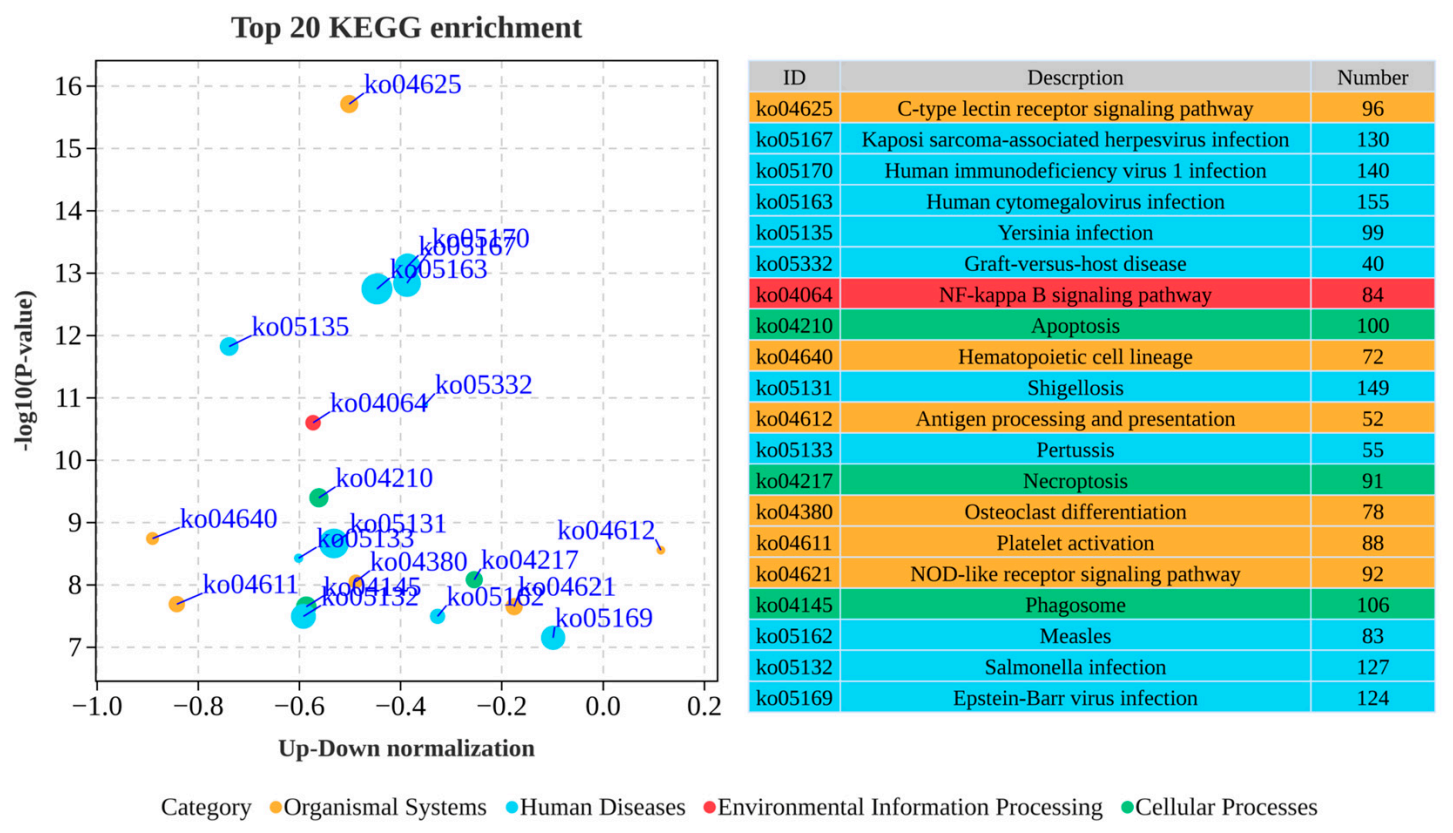
KEGG enrichment analysis of differentially expressed genes (DEGs). Distribution of the top 20 significantly enriched KEGG pathways (*p*-value < 0.05). Each dot represents a pathway, and dots in different colors indicate pathways belonging to different categories. In the left panel, the X-axis represents the up–down normalization value for each pathway, calculated as (number of upregulated DEGs-number of downregulated DEGs)/(total number of DEGs). A positive value (>0) indicates that upregulated genes predominate in the pathway, suggesting that the pathway as a whole may be activated or enhanced. The y-axis denotes pathway significance. The right panel displays the ID, description, and DEGs number of each pathway.

To further understand the biological functions of the DEGs, KEGG enrichment analysis was performed. The top 20 significantly enriched pathways are presented in Figure 4. Based on the value plotted on the X-axis, 95% of these pathways are below 0, indicating that the majority were broadly suppressed at the transcription level in the *V. harveyi*-infected group compared with the control group. Only the antigen processing and presentation pathway (ko04612) exhibited an activation status (>0). Notably, several critical immune pathways were markedly inhibited, including pathogen recognition pathways (C-type lectin receptor signaling pathway and NOD-like receptor signaling pathway), cell death pathways (apoptosis and necroptosis), phagosome, and the NF-κB signaling pathway. Collectively, these findings suggest a state of widespread transcriptional immune suppression in *V. harveyi*-infected erythrocytes.

### 3.4. Core Immune Pathways Identification and Interaction Network Analysis

**Figure 5:**
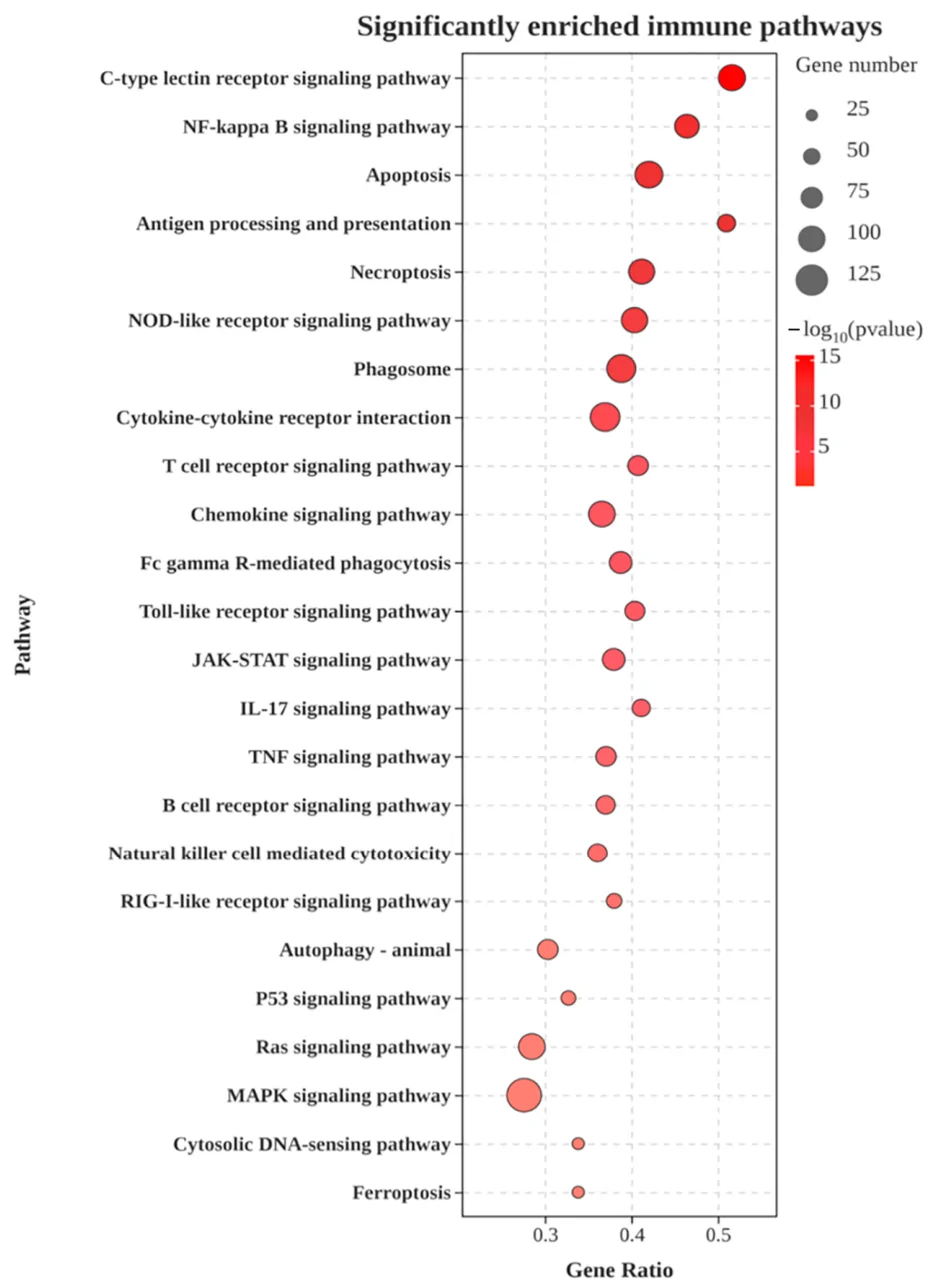
Twenty-four significantly enriched immune pathways are displayed above (*p*-value < 0.05). Circle size represents the number of enriched DEGs. Color intensity indicates the level of significance.

Based on the KEGG enrichment analysis, a total of 24 significantly enriched immune pathways are identified (*p*-value < 0.05) and displayed in Figure 5. These pathways included the C-type lectin receptor signaling pathway, NF-kappa B signaling pathway, apoptosis, antigen processing and presentation, necroptosis, NOD-like receptor signaling pathway, phagosome, cytokine–cytokine receptor interaction, T cell receptor signaling pathway, chemokine signaling pathway, Fc gamma R-mediated phagocytosis, toll-like receptor signaling pathway, JAK-STAT signaling pathway, IL-17 signaling pathway, TNF signaling pathway, B cell receptor signaling pathway, natural killer cell-mediated cytotoxicity, RIG-I-like receptor signaling pathway, autophagy–animal, p53 signaling pathway, Ras signaling pathway, MAPK signaling pathway, cytosolic DNA-sensing pathway, and ferroptosis, respectively.

**Figure 6:**
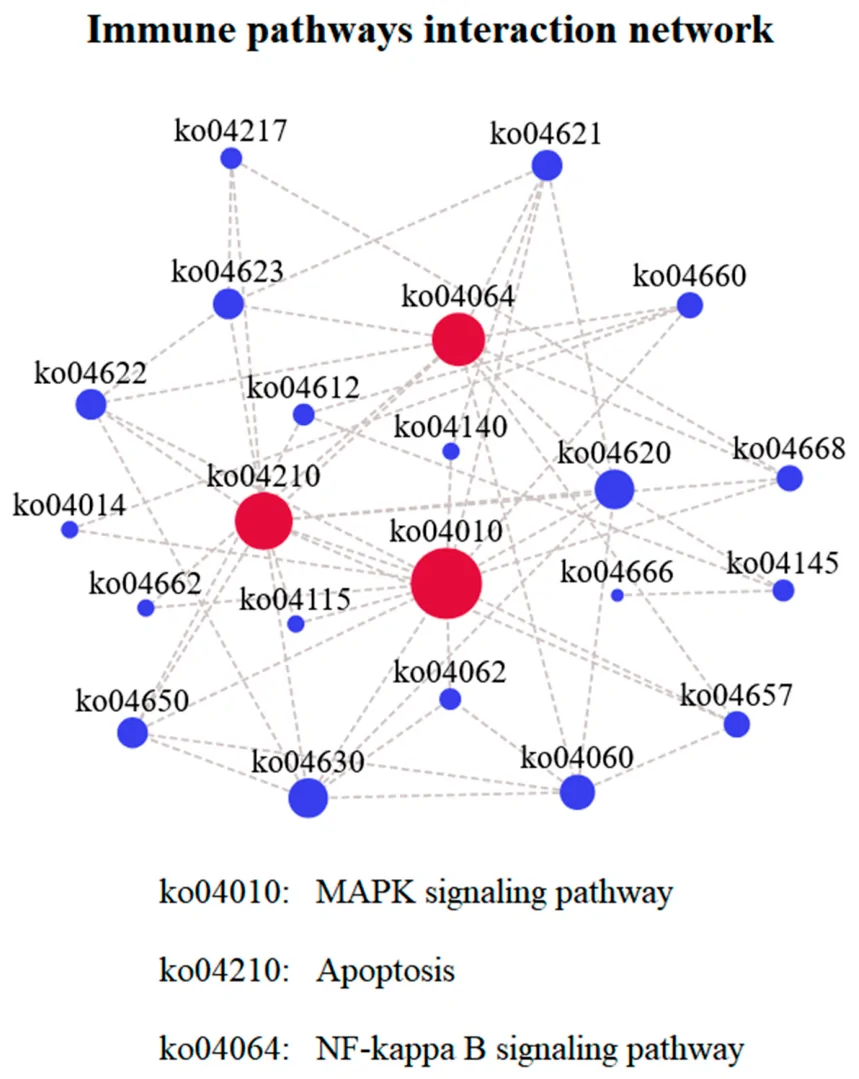
Identification of core immune pathways. An interaction network was constructed from the 24 significantly enriched immune pathways. Nodes denote pathways, and edges represent their interactions. Node size represents the degree of connectivity, with larger nodes indicating more central and important pathways. The top 3 core immune pathways are highlighted in red.

To identify core immune pathways, a pathway interaction network was constructed based on the hierarchical relationships in the KEGG database. The interaction degree of each pathway was calculated to evaluate its centrality within the network. As shown in Figure 6, 24 immune pathways exhibited extensive interconnections. Based on the interaction degree, the top three core immune pathways were identified as the MAPK signaling pathway (ko04010), apoptosis (ko04210), and the NF-kappa B signaling pathway (ko04064). For the MAPK signaling pathway, PPI network analysis revealed a tightly interacting network comprising 105 DEGs, of which 83.8% were significantly downregulated (Figure S3). The top three hub genes were *JUN* (PPI = 68), *TNF* (PPI = 63), and *MYC* (PPI = 60). For apoptosis, 75 DEGs constituted another densely linked network, of which 78.7% were markedly downregulated (Figure S4). The top three hub genes were *CASP3* (PPI = 55), *BCL2* (PPI = 53), and *ACTB* (PPI = 49). For the NF-κB signaling pathway, 51 DEGs formed a tightly interconnected network, of which 78.4% were significantly downregulated (Figure S5). The top three hub genes were *NFKB1* (PPI = 41), *TNF* (PPI = 40), and *IL-1β* (PPI = 34). Collectively, these findings were consistent with the KEGG enrichment results and further confirmed that *V. harveyi* infection induces widespread immune suppression in erythrocytes at the transcriptional level.

### 3.5. Heatmap Analysis of DEGs Involved in Core Immune Pathways

**Figure 7:**
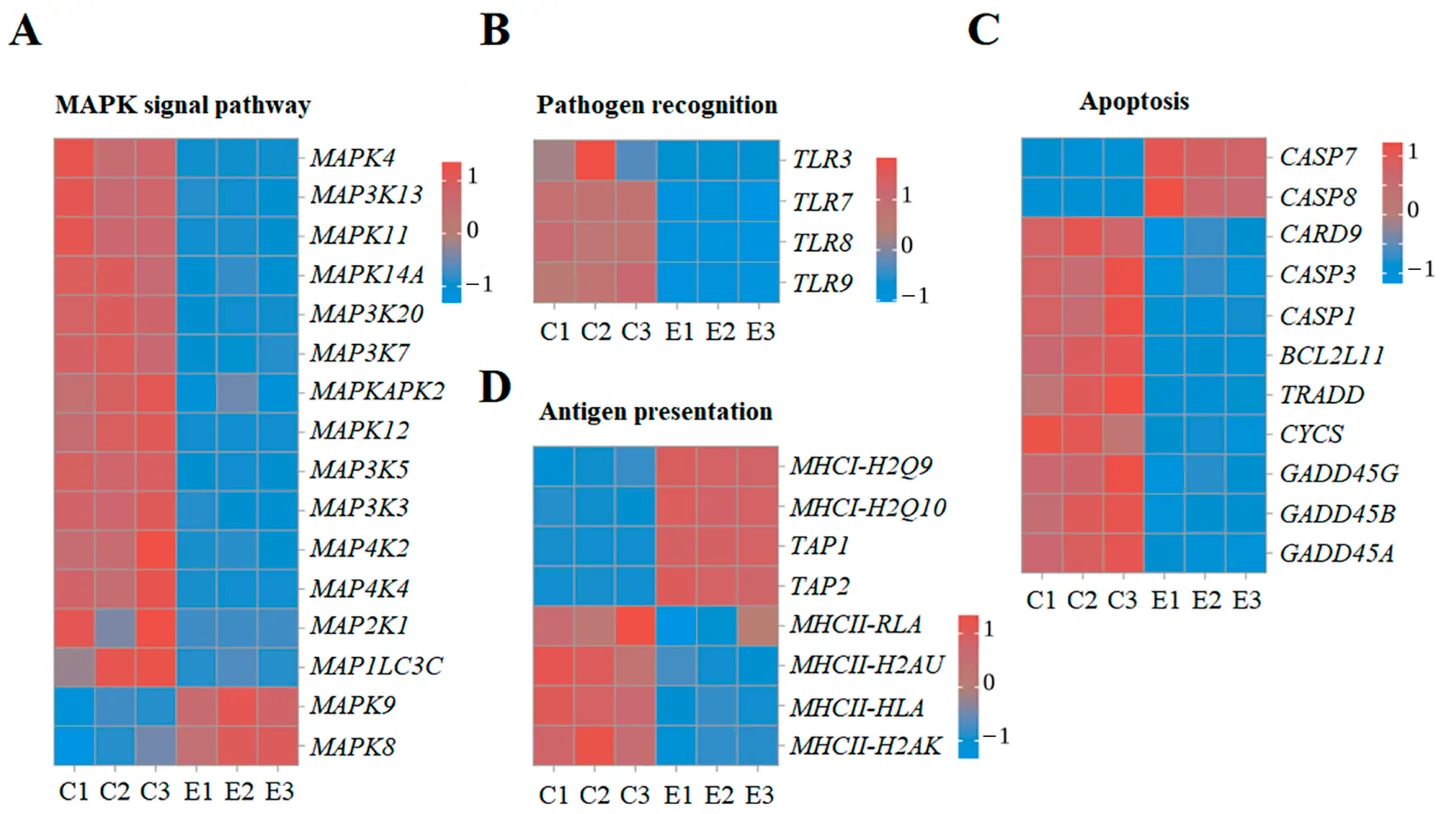
Heatmap analysis of representative differentially expressed genes (DEGs) involved in the MAPK signaling pathway (**A**), pathogen recognition (**B**), antigen presentation (**C**), and apoptosis (**D**). The displayed DEGs were chosen based on their functional relevance, such as MAPK kinases for the MAPK pathway and caspases for apoptosis.

**Figure 8:**
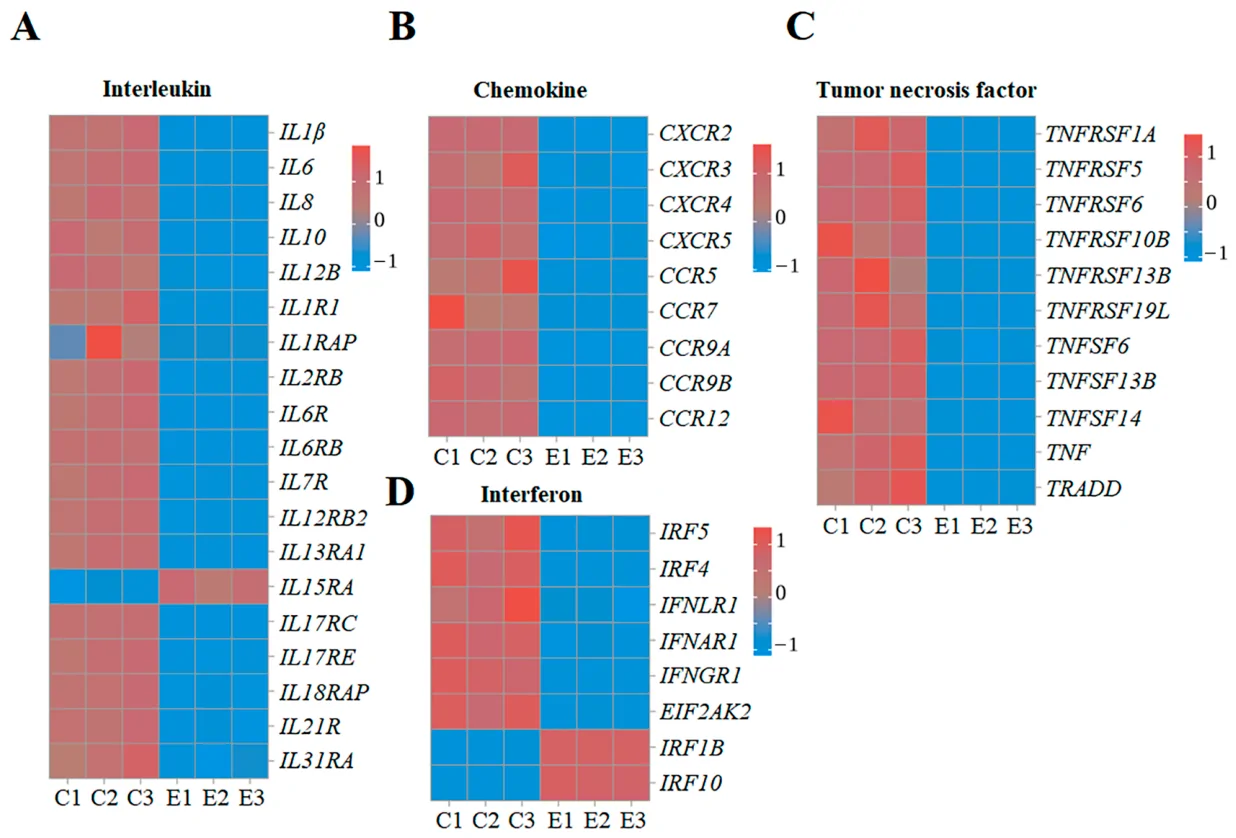
Heatmap analysis of representative differentially expressed genes (DEGs) associated with the NF-kappa B signaling pathway, including interleukin (**A**), chemokine (**B**), tumor necrosis factor (**C**), and interferon (**D**). The displayed downstream effectors were chosen based on their functional relevance to the NF-kappa B signaling pathway.

To determine the expression profiling of DEGs within each core immune pathway, heatmap analysis was performed. In the MAPK signaling pathway, except for *MAPK8/9,* most MAPK kinase-related DEGs were suppressed, including *MAP3K13*, *MAP3K7*, *MAPKAPK2*, *MAP3K3*, *MAP1LC3C*, *MAP4K4*, *MAPK14A*, *MAP4K2*, *MAP3K20*, *MAP3K5*, *MAPK11*, *MAPK4*, *MAP2K1*, and *MAPK12*, respectively (Figure 7A). Similarly, most apoptosis-related DEGs were significantly downregulated except for *CASP7/8*, including *CARD9*, *CASP3*, *CASP1*, *BCL2L11*, *TRADD*, *CYCS*, *GADD45A*, *GADD45B*, and *GADD45G*, respectively (Figure 7C). Notably, pathogen recognition receptors (*TLR3/7/8/9*) (Figure 7B) and *MHC-II* genes (*MHCII-RLA*, *MHCII-H2AU*, *MHCII-HLA*, and *MHCII-H2AK*) (Figure 7D) were significantly downregulated, whereas *MHC-I* genes (*MHCI-H2Q9* and *MHCI-H2Q10*) were upregulated. For the NF-kappa B signaling pathway, interleukin (Figure 8A), chemokine (Figure 8B), tumor necrosis factor (Figure 8C), and interferon (Figure 8D) were also extensively downregulated, with all chemokine and TNF genes showing reduced expression. Collectively, these findings provided transcriptional evidence for the novel strategy employed by *V. harveyi* to regulate host immune responses in erythrocytes.

## 4. Discussion

In this study, we investigated the interaction between *Vibrio harveyi* and leopard coral grouper erythrocytes, with a particular focus on bacterial uptake, global transcriptional responses, and the involvement of core immune pathways. Our findings demonstrate that leopard coral grouper erythrocytes are not merely passive oxygen carriers but active participants in innate immune defense, capable of internalizing bacterial pathogens and mounting complex transcriptional responses.

It is well known that endocytosis is a fundamental cellular process mediated by distinct molecular pathways [14]. However, whether fish erythrocytes exhibit endocytic activity toward pathogens remains poorly characterized. In this study, our findings demonstrated that *P. leopardus* erythrocytes can actively internalize *V. harveyi* in a bacterial dose-dependent manner, with comparable uptake efficiencies observed for both live and dead bacteria. Given that inactivated bacteria lack invasive capability, these results indicate an active, host-driven endocytic process rather than passive bacterial entry. This finding aligns with a previous report that flounder erythrocytes exhibited a marked capacity to engulf both live and dead *Edwardsiella tarda* [15]. Notably, unlike *Edwardsiella tarda*, which is a facultative intracellular pathogen, *V. harveyi* is generally recognized as an extracellular bacterium that primarily exerts its virulence through secreted exotoxins and proteases [24]. This fundamental difference in infection strategy provides a plausible explanation for the comparable internalization efficiencies of live and dead *V. harveyi* observed in this study. Similar observations were found in grass carp (*Ctenopharyngodon idella*) and Asian catfish (*Clarias fuscus*), whose erythrocytes have been documented to internalize *Aeromonas hydrophila*, *Staphylococcus aureus*, and *Escherichia coli* [25,26]. These collective data suggest that erythrocyte-mediated bacterial internalization may represent a conserved immune function across diverse teleost species.

*V. harveyi* is a Gram-negative pathogen responsible for severe vibriosis in teleost [24]. Previous studies in fish cell lines have shown that its hemolysin and T3SS activate caspase-3 and induce apoptosis at the functional level [27,28]. For example, hemolysin has been shown to induce apoptosis in flounder gill cells through caspase-3 activation, as evidenced by membrane protrusions, chromatin condensation, and apoptotic body formation [27]. Likewise, the T3SS system rapidly caused carp fathead minnow cell rounding, DNA fragmentation, and caspase-3 activation [28]. In contrast, our transcriptomic data revealed that the apoptosis pathway was significantly inhibited at the transcriptional level in *V. harveyi*-infected erythrocytes. Pathogen recognition pathways (C-type lectin receptor and NOD-like receptor signaling), necroptosis, phagosome, and the NF-κB pathway were also all significantly inhibited at the transcriptional level. These findings suggest that *V. harveyi* induces cell type-specific responses, with transcriptional immune inhibition predominating in erythrocytes. To identify the core immune pathways underlying this transcriptional immunosuppression, a pathway interaction network was constructed based on the 24 significantly enriched immune pathways. Three core pathways based on the interaction degree emerged and were identified: the MAPK signaling pathway, apoptosis, and the NF-κB signaling pathway. Within each of these pathways, the majority of DEGs were significantly downregulated with 83.8% in MAPK signaling, 78.7% in apoptosis, and 78.4% in NF-κB signaling.

The MAPK signaling pathway is a central regulator of innate immune responses and is composed of three major subfamilies: ERK, JNK, and p38 MAPK [29]. In this study, most MAPK kinase-related DEGs were significantly inhibited at the transcriptional level, and the three hub genes (*JUN*, *TNF*, and *MYC*) within this pathway were all significantly downregulated. This pattern is consistent with findings in abalone hemocytes, where pathogenic *V. harveyi* ORM4 strain delayed p38 phosphorylation to avoid immune clearance [30]. However, the non-pathogenic *V. harveyi* LMG 7890 strain induces rapid p38 activation, leading to phagocytosis and high ROS production, which eliminate the bacteria. These findings demonstrate that pathogenic *V. harveyi* actively interferes with p38-MAPK signaling to avoid host immune clearance. However, our study reveals a transcriptional suppression of multiple MAPK components in erythrocytes, suggesting that the suppression in erythrocytes may be more broad-spectrum. This difference may reflect that erythrocytes lack specialized antimicrobial machinery and are more susceptible to transcriptional shutdown of signaling pathways. Additionally, a study in pearl gentian grouper revealed that *V. harveyi* infection suppressed JNK pathway while enhancing the ERK pathway [31], indicating that *V. harveyi* may differentially modulate distinct branches of the MAPK cascade to favor its own survival.

Apoptosis is a fundamental host defense mechanism that eliminates infected cells and restricts pathogen dissemination [32]. In this study, apoptosis-related genes were predominantly downregulated in *V. harveyi*-infected erythrocytes. Notably, *CASP3*, a key executioner caspase and critical pro-apoptotic regulator, was significantly downregulated at the transcriptional level. Previous studies reported that *V. harveyi* induces caspase-3 activation and promotes apoptosis in flounder gill cells [27] and sea bream fibroblasts [33]. However, these observations were made in fibroblast and gill cell lines, whereas our study examined erythrocytes. Conversely, a study in *miiuy croaker* demonstrated that *V. harveyi* inhibits apoptosis through *ACKR4a*-induced autophagy [34], supporting cell type-specific strategies. Although the transcription of *CASP8* and *CASP7* was upregulated in this study, the significant downregulation of caspase-3 suggests that the apoptotic machinery of erythrocytes may be inhibited at the effector stage. However, the functional relevance of these transcriptional changes requires verification by measuring caspase enzyme activity. Additionally, the reduced expression of *CASP1* implies a concomitant suppression of inflammasome-mediated pyroptosis, indicating that *V. harveyi* may simultaneously inhibit multiple programmed cell death pathways in erythrocytes.

The NF-κB signaling pathway serves as a central regulator of innate immunity, orchestrating the production of pro-inflammatory cytokines, chemokines, and antimicrobial effectors [35]. In this study, 78.4% of DEGs in this pathway were downregulated, with hub genes (*NFKB1*, *TNF*, *IL-1β*) markedly suppressed. Previous study in *Miichthys miiuy* demonstrated that *V. harveyi* suppresses NF-κB signaling via *Zw10*-mediated MyD88 degradation [36]. *V. harveyi* also induced the expression of eukaryotic translation initiation factor 3k (*eIF3k*), and *eIF3k* functions as a specific suppressor of the MyD88-dependent NF-kB pathway [37]. Similarly, upregulating miR-214 induced by *Vibrio harveyi* subsequently inhibits the production of inflammatory cytokines by targeting MyD88 to avoid excessive inflammation [38]. These observations highlight the importance of MyD88, an upstream regulator of NF-kB pathway activation, in *V. harveyi*-induced immune inhibition. Consistent with these observations, *MyD88* expression in *V. harveyi*-infected erythrocytes was significantly downregulated by 3.86-fold compared with the control group. Moreover, pathogen recognition receptors *TLR3*, *TLR7*, *TLR8*, and *TLR9* were significantly downregulated in *V. harveyi*-infected erythrocytes. This finding is consistent with observations in spotted sea bass (*Lateolabrax maculatus*), where most TLR genes were downregulated following *V. harveyi* infection [39]. Notably, *TLR7*/*8*/*9* signals exclusively through the MyD88-dependent pathway to activate NF-κB, whereas *TLR3* signals through the MyD88-independent TRIF pathway. Therefore, the coordinated downregulation of *TLR7/8/9* and *MyD88* would disrupt MyD88-dependent TLR signaling, consequently diminishing the erythrocyte immune response mediated by the NF-κB pathway at the transcriptional level.

Collectively, our transcriptomic data reveal that *V. harveyi* infection induces transcriptional suppression of MAPK, NF-κB, and apoptotic signaling axes in leopard coral grouper erythrocytes. The coordinated downregulation of TLR receptors, MyD88, and cytokines provides transcriptional evidence for a multi-layered inhibition of host immune responses. The active endocytic uptake of *V. harveyi* by erythrocytes, coupled with transcriptional immune inhibition, suggests that erythrocytes may serve as a potential intracellular reservoir facilitating bacterial persistence. Beyond the mechanistic insights, our findings may have practical implications for disease management. The transcriptional profiles of erythrocyte immune genes could be developed as sentinel biomarkers for early warning of vibriosis in aquaculture. Furthermore, the core immune pathways identified in this study represent potential targets for immunostimulant or vaccine development aimed at enhancing erythrocyte-mediated immunity against Vibrio infections.

## 5. Conclusions

In summary, this study provides comprehensive evidence that leopard coral grouper erythrocytes are not merely passive oxygen carriers, but active participants in innate immune defense against *V. harveyi* infection. Our findings demonstrate that erythrocytes possess the capacity to actively internalize *V. harveyi* in a dose-dependent manner, establishing them as an important component of the host immune system. The transcriptomic landscape of infected erythrocytes reveals widespread transcriptional suppression and identified three core pathways: the MAPK signaling pathway, apoptosis, and the NF-κB signaling pathway. The coordinated downregulation of TLR receptors, *MyD88*, and cytokines (interleukins, chemokines, tumor necrosis factors, and interferons) provides transcriptional evidence for a multi-layered strategy employed by *V. harveyi* to regulate host immune responses in erythrocytes. These findings provide novel insights into the immune functions of teleost erythrocytes during bacterial infection.

## Figures and Tables

**Table 1:** Quality of RNA sequencing data. C, control group; E, *V. harveyi*-infected group. Each group consisted of three biological replicates, denoted as -1, -2, and -3.

Samples	Raw Reads	Clean Reads	Clean Read Ratio (%)	Clean Reads (Ribosome Depletion)	Mapped Reads	Mapped Read Ratio (%)
C-1	44,896,468	44,665,636	99.49	44,625,124	39,133,534	87.69
C-2	47,997,052	47,783,590	99.56	47,742,840	41,775,774	87.50
C-3	54,953,972	54,684,030	99.51	54,636,846	47,770,315	87.43
E-1	40,004,030	39,758,238	99.39	39,739,748	32,937,863	82.88
E-2	46,740,032	46,428,102	99.33	46,397,114	38,546,892	83.08
E-3	53,086,546	52,700,200	99.27	52,665,258	43,495,312	82.59

## Data Availability

All data used in this study are presented in this article.

## Supplementary Materials

The following supporting information can be downloaded at https://www.mdpi.com/article/10.3390/ani16182831/s1, Table S1. List of the primers used for qRT-PCR. Figure S1. Microscopic observation of purified Leopard coral grouper erythrocytes. Figure S2. Validation of randomly selected DEGs by qRT-PCR compared with RNA-Seq data. The relative expression levels of 10 DEGs were quantified via qRT-PCR, and the results were then compared with the corresponding RNA-seq data. Figure S3. Interaction network was constructed from differentially expressed genes (DEGs) involved in the MAPK signaling pathway. The nodes in the network denote DEGs, and the lines connecting different nodes denote interactions between the DEGs. Figure S4. Interaction network was constructed from differentially expressed genes (DEGs) involved in the apoptosis pathway. The nodes in the network denote DEGs, and the lines connecting different nodes denote interactions between the DEGs. Figure S5. Interaction network as constructed from differentially expressed genes (DEGs) involved in the NF-kappa B signaling pathway. The nodes in the network denote DEGs, and the lines connecting different nodes denote interactions between the DEGs.

## Abbreviations

The following abbreviations are used in this manuscript:

DEGs	Differentially expressed genes
MAPK	Mitogen-activated protein kinase
PRR	Pattern recognition receptor
PAMP	Pathogen-associated molecular pattern
TLR	Toll-like receptor
NOD2	Nucleotide-binding oligomerization domain 2
NLRX1	NLR family member X1
NLRC3	NLR family CARD domain containing 3
NLRC5	NLR family CARD domain containing 5
PBS	Phosphate-buffered saline
FITC	Fluorescein isothiocyanate
MOI	Multiplicity of infection
FPKM	Fragments per kilobase of transcript per million mapped reads
IL-17	Interleukin-17
TNF	Tumor necrosis factor
RIG-I	Retinoic acid-inducible gene I
PPI	Protein–protein interaction
JUN	Jun proto-oncogene
MYC	Myc proto-oncogene protein
CASP3	Caspase-3
BCL2	B-cell lymphoma 2
ACTB	Actin beta
NFKB1	NF-kappa-B p105 subunit
IL-1β	Interleukin-1 beta
ERK	Extracellular signal-regulated kinase
JNK	c-Jun N-terminal kinase
MyD88	Myeloid differentiation primary response protein
CASP7	Caspase-7
CASP8	Caspase-8
CARD9	Caspase recruitment domain-containing protein 9
TRADD	Tumor necrosis factor receptor type 1-associated DEATH domain protein
CYCS	Cytochrome c
GADD45	Growth arrest and DNA damage-inducible protein
MHC	Major Histocompatibility Complex
