# Supplementary material for: Transcriptome Analysis of Plectropomus leopardus Erythrocytes Reveals Transcriptional Immune Suppression Induced by Vibrio harveyi Infection
Source: Animals (Basel). 2026 Sep 9;16(18):2831. doi: 10.3390/ani16182831 (PMC13603331; doi:10.3390/ani16182831)
Supplement: Supplementary file 1 [file animals-16-02831-s001.zip › animals-4505422-supplementary.pdf]

Table S1. List of the primers used for qRT-PCR.

Gene name	Gene ID	Forward primer (5′-3′)	Reverse primer (5′-3′)
TAP2	XM_042506860.1	GAGAAGAAGCAGAAAGCCAGAG	GCCAGTGTAGAAGGGAATGAAC
IRF1B	XM_042498721.1	AGGGAGCCAGTGGAGTGAAT	CGTCTGTGGGATTTGTGAGG
MHCI-H2Q10	XM_042506463.1	AGCGAGTGCGTGGATTGGTT	GCCGTCGTCTTCAGTCAGGATA
CYCS	XM_042494604.1	CATACTGTGGAGAATGGTGGAA	TTTGTTGGCATCAGTGTAGGAG
CARD9	XM_042481476.1	AGAGCCAGGAGTGTTGAGGAGA	CTGTCGGAGGGTTTGTTTGTTT
GADD45G	XM_042488101.1	TTCACCCTCATCCAGGCTTTC	AGTCATACACGCTGCGGCTCT
CTSZ	XM_042505676.1	AAGAAGCAGCCGTGTTATGTCC	CCATTGATGTTTCTCCAGTCCC
CTSA	XM_042505604.1	GGGGAAGATTTATGGCGAGAC	GCCACCCTCACAATCCAAGTA
CXCR4	XM_042494459.1	GCACATCCTGGTGGGCTTCA	AGGGCTTTCTTCTTCAGAGTTTGG
MAP3K7	XM_042500834.1	TGTCCAGGGTGTCCAGCGTAG	GCGGCTGACTTGTAACTGGTGAT
β-actin	OK483033.1	CATCCTGCGTCTGGACTTGG	TCTCGGCTGTGGTGGTGAAA

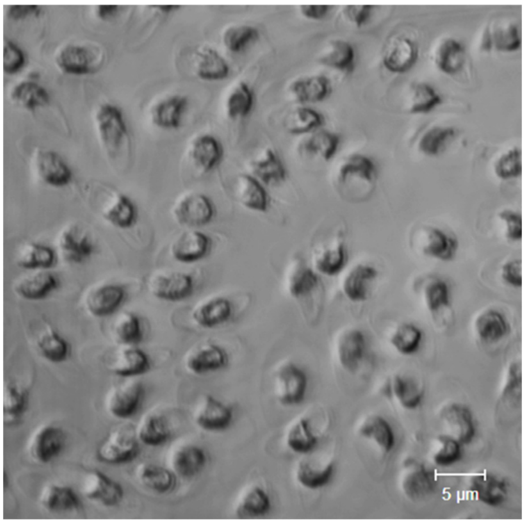

Figure S1. Microscopic observation of purified Leopard coral grouper erythrocytes.

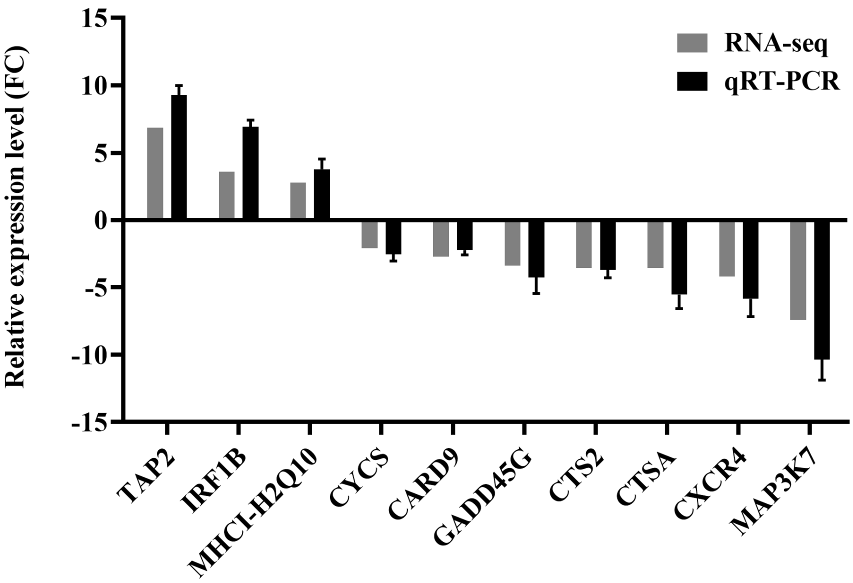

Figure S2. Validation of randomly selected DEGs by qRT-PCR compared with RNA-Seq data. The relative expression levels of 10 DEGs were quantified via qRT-PCR, and the results were then compared with the corresponding RNA-seq data.

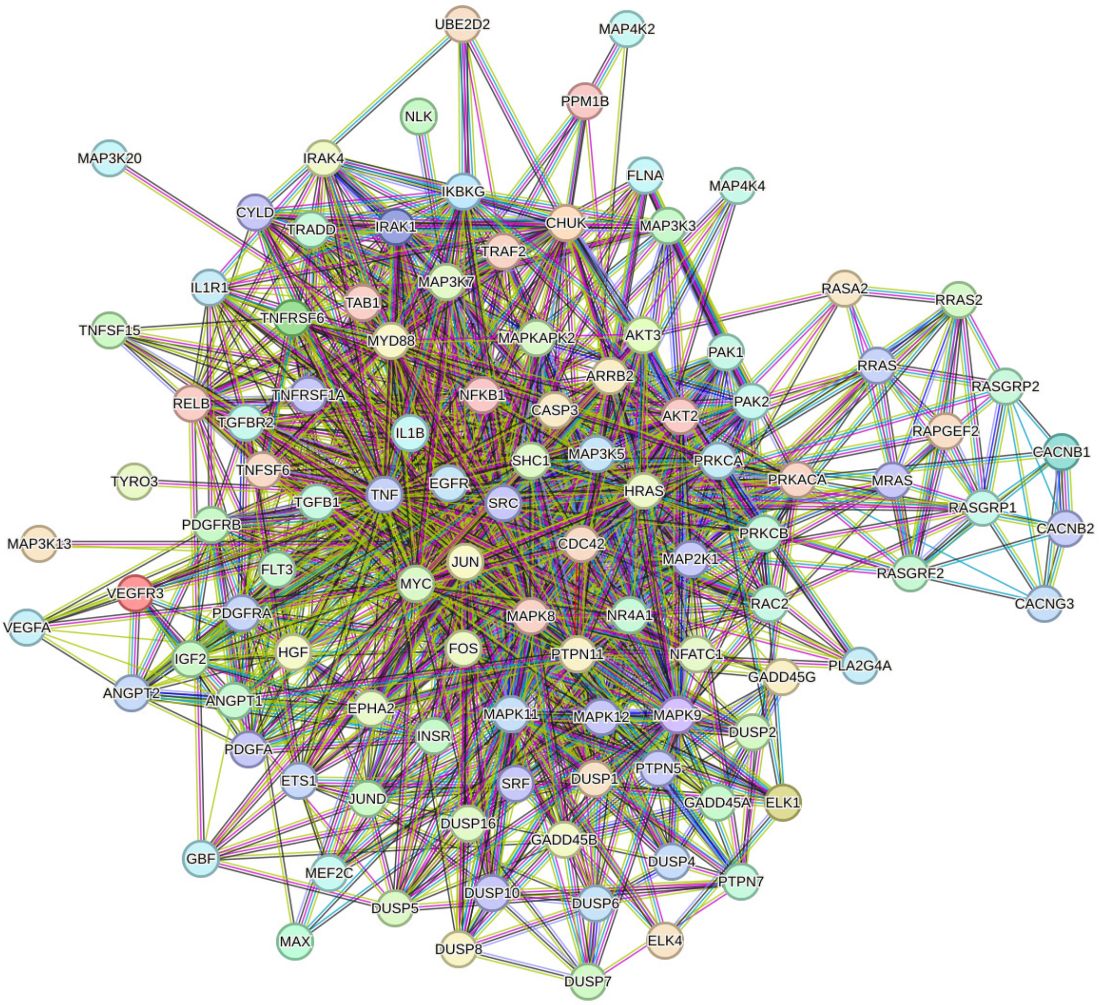

Figure S3. Interaction network was constructed from differentially expressed genes (DEGs) involved in the MAPK signaling pathway. The nodes in the network denote DEGs, and the lines connecting different nodes denote interactions between the DEGs.

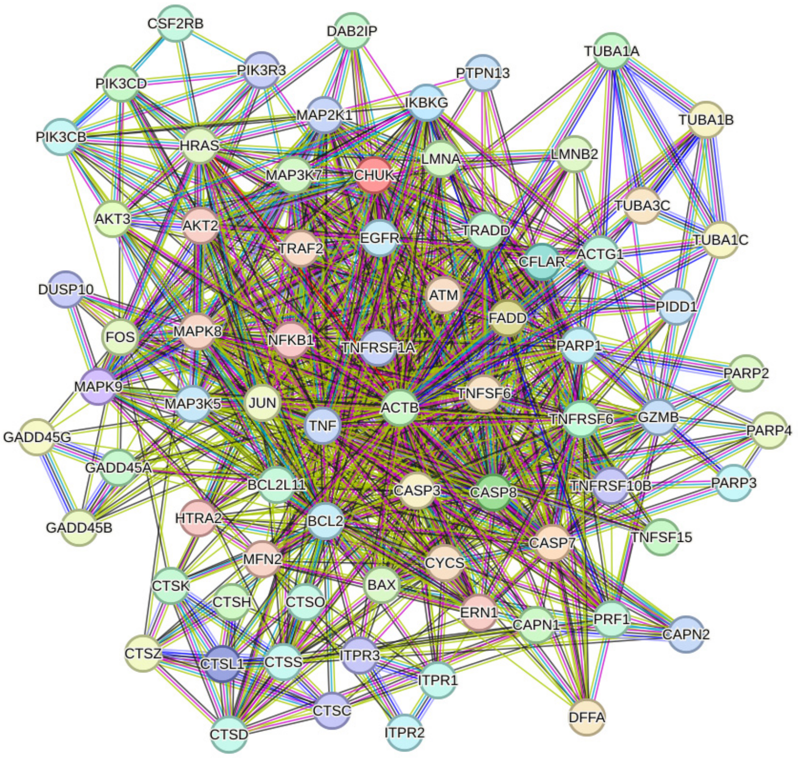

Figure S4. Interaction network was constructed from differentially expressed genes (DEGs) involved in the apoptosis pathway. The nodes in the network denote DEGs, and the lines connecting different nodes denote interactions between the DEGs.

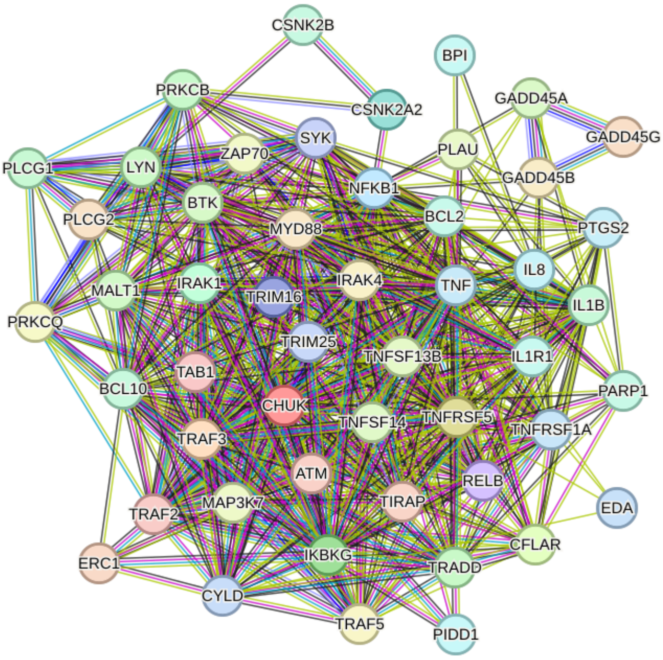

Figure S5. Interaction network as constructed from differentially expressed genes (DEGs) involved in the NF-kappa B signaling pathway. The nodes in the network denote DEGs, and the lines connecting different nodes denote interactions between the DEGs.
